# Supplementary material for: What do people think of intuitive eating? A qualitative exploration with rural Australians
Source: PLoS One. 2023 Aug 17;18(8):e0278979. doi: 10.1371/journal.pone.0278979 (PMC10434910; doi:10.1371/journal.pone.0278979)
Supplement: Attachment — Submitted filename: Response to Reviewer Comments_3Jul2023.docx [file pone.0278979.s002.docx]

Response to Reviewer Comments

What do people think of intuitive eating?  A qualitative exploration with rural Australians

Reviewer #1: I thank the authors for addressing all of my previous comments. I found a couple of typographical errors the authors may want to fix should the manuscript be accepted for publication (line numbers refer to track changes version):
Line 62: BMI should be written out in full first here, then on lines 78 and 83 just use the abbreviation rather than spelling out in full again.

Author response: This correction has been made.

Line 114 missing ) after (e.g., [13-15

Author response: This correction has been made.

Line 598 is ‘(41)’ a reference? It differs in style to the other in-text citations which use square brackets"

Author response: This correction has been made. Please note that the correct reference number is 42. This correction has been made.
